# Positive end-expiratory pressure increases intracranial pressure but not pressure reactivity index in supine and prone positions: a porcine model study

**DOI:** 10.3389/fmed.2024.1501284

**Published:** 2025-01-07

**Authors:** Rønnaug Hammervold, Erta Beqiri, Peter Smielewski, Benjamin S. Storm, Erik W. Nielsen, Claude Guérin, Shirin K. Frisvold

**Affiliations:** ^1^Department of Anaesthesia and Intensive Care, Nordland Hospital Trust, Bodø, Norway; ^2^Department of Clinical Medicine, Faculty of Health Sciences, UiT The Arctic University of Norway, Tromsø, Norway; ^3^Research Laboratory, Nordland Hospital Trust, Bodø, Norway; ^4^Brain Physics Laboratory, Department of Clinical Neurosciences, University of Cambridge, Cambridge, United Kingdom; ^5^Faculty of Nursing and Health Sciences, Nord University, Bodø, Norway; ^6^Faculty of Medicine, Institute of Clinical Medicine, University of Oslo, Oslo, Norway; ^7^Faculté de médecine Lyon-Est, Université de Lyon, Lyon, France; ^8^Department of Anaesthesia and Intensive Care, University Hospital of North Norway, Tromsø, Norway

**Keywords:** cerebral autoregulation, ICP, neurocritical care, neuromonitoring, pulmonary issues, ventilation, mechanical ventilation, transpulmonary pressure

## Abstract

**Introduction:**

Positive end-expiratory pressure (PEEP) and prone positioning can improve gas exchange by promoting uniform lung aeration. However, elevated ventilation pressures may increase intracranial pressure (ICP) and disrupt cerebral autoregulation. This study investigated the effects of PEEP on ICP and cerebral autoregulation in a porcine model with healthy lungs and normal ICP, comparing prone and supine positions. Cerebral autoregulation was assessed through cerebrovascular reactivity using the pressure reactivity index (PRx). We also explored whether other baseline variables influenced potential variances in ICP and PRx.

**Methodology:**

Twelve anesthetized pigs were randomized to begin in either supine or prone position, across PEEP of 5, 10, 15, and 20 cmH_2_O. Continuous monitoring included esophageal pressure to calculate end-inspiratory and end-expiratory transpulmonary pressures. The ICM+^®^ software (University of Cambridge Enterprise, Cambridge, United Kingdom) was used for high-resolution data collection, signal processing and ICP curve analysis. Linear mixed-effects models and ANOVA were used to analyze changes in ICP and PRx and the influence of position. An exploratory correlation analysis was conducted on baseline variables potentially related to the ICP increase.

**Results:**

Mean ICP increase was 1.0 mmHg ± 0.9 at 10 cmH_2_O PEEP, 2.0 mmHg ± 1.7 at 15 cmH_2_O PEEP, and 3.1 mmHg ± 1.6 at 20 cmH_2_O PEEP compared to a baseline PEEP of 5 cmH_2_O (*p* < 0.001). The effect of PEEP increase on ICP was not influenced by body position. PRx remained unaffected by PEEP. PEEP-induced increases in ICP were higher in cases of higher baseline ICP, higher central venous pressure, lower respiratory system elastance and lower end-inspiratory and end-expiratory transpulmonary pressures.

**Conclusion:**

Increasing PEEP elevates ICP regardless of body position without adversely affecting cerebral autoregulation in a healthy porcine model. Baseline ICP, central venous pressure, respiratory system elastance and end-inspiratory and end-expiratory transpulmonary pressure may influence the magnitude of ICP changes.

## 1 Introduction

Invasive positive pressure mechanical ventilation (IPPMV) is a life-saving procedure in patients with acute respiratory failure or severe brain injury, as long as the negative consequences that its settings can entail are minimized (1). To prevent lung injury, lung protective ventilation (LPV) aims to preserve the lung parenchyma from overdistension by reducing tidal volume (VT) and promote homogenisation of aerated lung mass by a judicious level of positive end-expiratory pressure (PEEP) and prone positioning (2–5).

End-inspiratory transpulmonary pressure (TPP_ei_) represents the stress imposed on the lung parenchyma and is generated by PEEP levels and VT (6). Although the effects of IPPMV on the heart-lung interaction have been extensively studied, the complex interplay with other key organs should also be considered to ensure overall safety (7). Among these, the brain is of paramount importance as IPPMV can have deleterious effects on cerebral perfusion, leading to a delay in full recovery in the short-term and neurological sequalae in survivors of brain injury in the long term (8). Although the impact of IPPMV on cerebral perfusion has been studied to some extent, little attention has been paid to the specific effects of PEEP combined with prone position on intracranial pressure (ICP) and cerebral autoregulation (CA) (9). CA has been suggested as a parameter that may guide cerebral perfusion pressure (CPP) targets (10). New tools have emerged to study CA, which represents a crucial defence mechanism for maintaining cerebral blood perfusion, in case of acute changes in perfusion pressure, oxygen supply, carbon dioxide, venous return, or cardiac output. A key method for identifying disturbances in CA is the pressure reactivity index (PRx), which uses the moving correlation coefficient between slow changes in arterial blood pressure (ABP) and ICP (11–13). Negative PRx values signify preserved autoregulation, whereas positive values indicate impairment (14). In addition to PRx, the compensatory reserve index (RAP) is used to evaluate intracranial compliance, where low values indicate sufficient compensatory reserve, and values approaching one reflect reduced compliance (15). PRx has been validated in porcine models (16), but the correlation between PRx, PEEP and body position changes has not been studied previously. Consequently, with the aim of investigating how changes in PEEP in the prone and supine positions affect ICP and PRx in a porcine model with healthy lungs and normal ICP, the present study is the first step in a comprehensive investigation of the lung-brain cross talk in normal as well as diseased condition.

## 2 Materials and methods

Following the approval of The Norwegian Animal Research Authority (FOTS ID 27107), we conducted a prospective, randomized, controlled animal study. The study was conducted in strict compliance with the Norwegian Laboratory Animal Regulations and the EU Directive 2010/64/EU to ensure the humane treatment of animals. We confirm the use of the ARRIVE guidelines checklist for the reporting of animal research, as appropriate.

### 2.1 Animal preparation

Fifteen Norwegian domestic landrace pigs (*Sus scrofa domesticus*) were studied. Twelve pigs, comprising eleven males and one female, were included in the study, with an average weight of 25 kg (range 23–28 kg). Two were excluded due to infection and hypotension, and one was excluded due to technical difficulties with ICP monitoring. The animals were retrieved from a local farm. Immediately after being removed from the pen, they were sedated with an intramuscular injection of 20 mg/kg ketamine, 0.5 mg/kg midazolam, and 1 mg atropine. The animals were directly transported to the animal research facility (ANILAB) at the North University of Bodø. Transportation time was approximately 10 min.

On arrival, they were identified by unique identifiers (e.g., sgb307, sgb311). Bilateral auricular veins were cannulated. Anesthesia was induced with an intravenous infusion comprising morphine (2 mg/kg/h), midazolam (0.15 mg/kg/h), and pentobarbital (4 mg/kg/h). The animals, positioned prone, were intubated with a Portex ID 6.5 to 7.0-mm-inner diameter endotracheal tube from Smiths Medical International Ltd. (Kent, United Kingdom). All animals were regularly checked for the pedal withdrawal reflex, palpebral reflex, and jaw tone, following standard protocol, to ensure that the depth of anesthesia was always adequate.

Mechanical ventilation was initiated using a Datex-Ohmeda Engström Carestation intensive care ventilator (GE Healthcare, Madison, WI) set to a volume-control mode with a VT of 9 mL/kg, fraction of oxygen in air (FiO_2_) of 0.40, PEEP of 5 cmH_2_O, inspiratory-to-expiratory time ratio 1:2, inspiratory pause 20%, keeping the respiratory rate between 20–30 breaths/min to maintain adequate ventilation with PaCo_2_ levels within the range of 35–45 mmHg.

A FluxMed esophageal catheter (MBMED, Martinez, Argentina) was placed and connected to the ventilator to monitor the esophageal pressure (Pes). The correct placement of the probe into the esophagus was ascertained by a ∆Pes/∆Paw ratio close to unity (0.8–1.2) where DPes and DPaw are the change in Pes and airway pressure (Paw), respectively during an expiratory hold (17).

After placing the animals in the supine position, we inserted a MAC^™^ two-lumen central venous catheter (CVC) with an integrated hemostasis valve (ARROW, Morrisville, United States) via ultrasound guidance into the right external jugular vein. Through this valve, we advanced a 7.5 Fr. Swan-Ganz pulmonary artery catheter (Edwards Lifesciences Corporation, Irvine, CA). Using a sterile cut-down technique, a 4 Fr., 8 cm PiCCO thermodilution catheter (Pulsion/Getinge, Gothenburg, Sweden) was placed in the left femoral artery. These catheters were connected to dedicated pressure transducers and a thermodilution cardiac output monitoring system (Edwards Lifesciences Corporation), interfaced with the Intellivue MP70 Monitor (Philips Healthcare, Cambridge, CA).

To ensure euvolemia, an intravenous fluid bolus of 500 mL of Ringer acetate (Fresenius Kabi, Oberdorf, Switzerland) was administered at a rate of 30 mL/kg/h, followed by a 5 mL/kg/h continuous infusion throughout the study.

A suprapubic catheter was placed via cystotomy for urinary drainage.

### 2.2 Cerebral monitoring

Subsequent instrumentation in the prone position included the insertion of a Codman MicroSensor ICP Transducer (Medos International SÀRL, Le Locle, Switzerland) into the left frontal hemisphere through a burr hole. The transducer was connected to a Codman ICP Express monitor (Codman & Shurtleff, MA, United States) for real-time ICP monitoring.

### 2.3 Physiological monitoring and data acquisition

The MP70 Monitor continuously monitored electrocardiography, oxygen saturation via plethysmography (SpO_2_), end-tidal carbon dioxide (EtCO_2_), and body temperature. Mean invasive arterial (ABP), central venous (CVP), and pulmonary artery blood pressures (PAP), as well as cardiac output (CO), were continuously measured. Extravascular lung water (EVLW) and stroke volume variation (SVV) were measured intermittently by thermodilution according to the PICCO manufacturer’s guidelines. The ICP signal from the Codman monitor was integrated into the MP70. Respiratory variables were collected by the Engström Carestation ventilator (see below).

**Figure 1:**
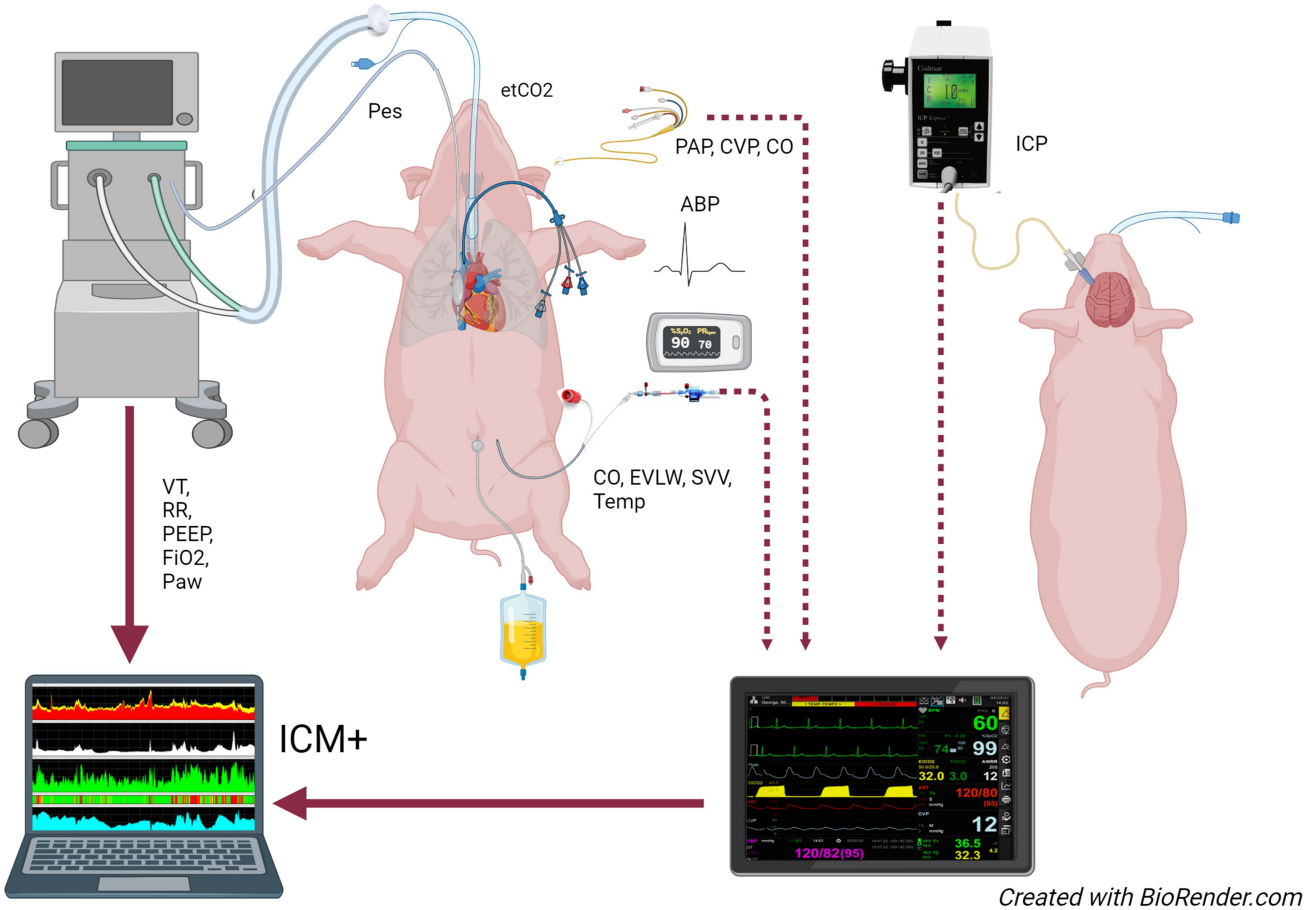
Study setup. The multiparametric monitor and the ventilator are connected to a computer running ICM+ software for data acquisition. ABP, arterial blood pressure; CO, cardiac output; CVP, central venous pressure; etCO_2_, end-tidal partial pressure of CO_2_; EVLW, extravascular lung water; FiO_2_, fraction of inspired oxygen; ICP, intracranial pressure; PAP, pulmonary artery pressure; Paw, airway pressure; Pes, esophageal pressure; PEEP, positive end-expiratory pressure; RR, respiratory rate; SVV, stroke volume variation; Temp, temperature; VT, tidal volume.

The Intensive care monitoring software (ICM+^®^, University of Cambridge Enterprise, Cambridge, United Kingdom) was used for signal acquisition, waveform analysis, and data summaries. High-resolution data from the MP70 and the ventilator were streamed in real-time to a laptop running the software, providing integration and synchronization of cerebral, respiratory, and hemodynamic variables, as shown in Figure 1.

Arterial blood was drawn at each PEEP level throughout the experiment to measure pH, partial pressure of oxygen (PaO_2_), and carbon dioxide (PaCO_2_).

### 2.4 Study procedure

**Figure 2:**
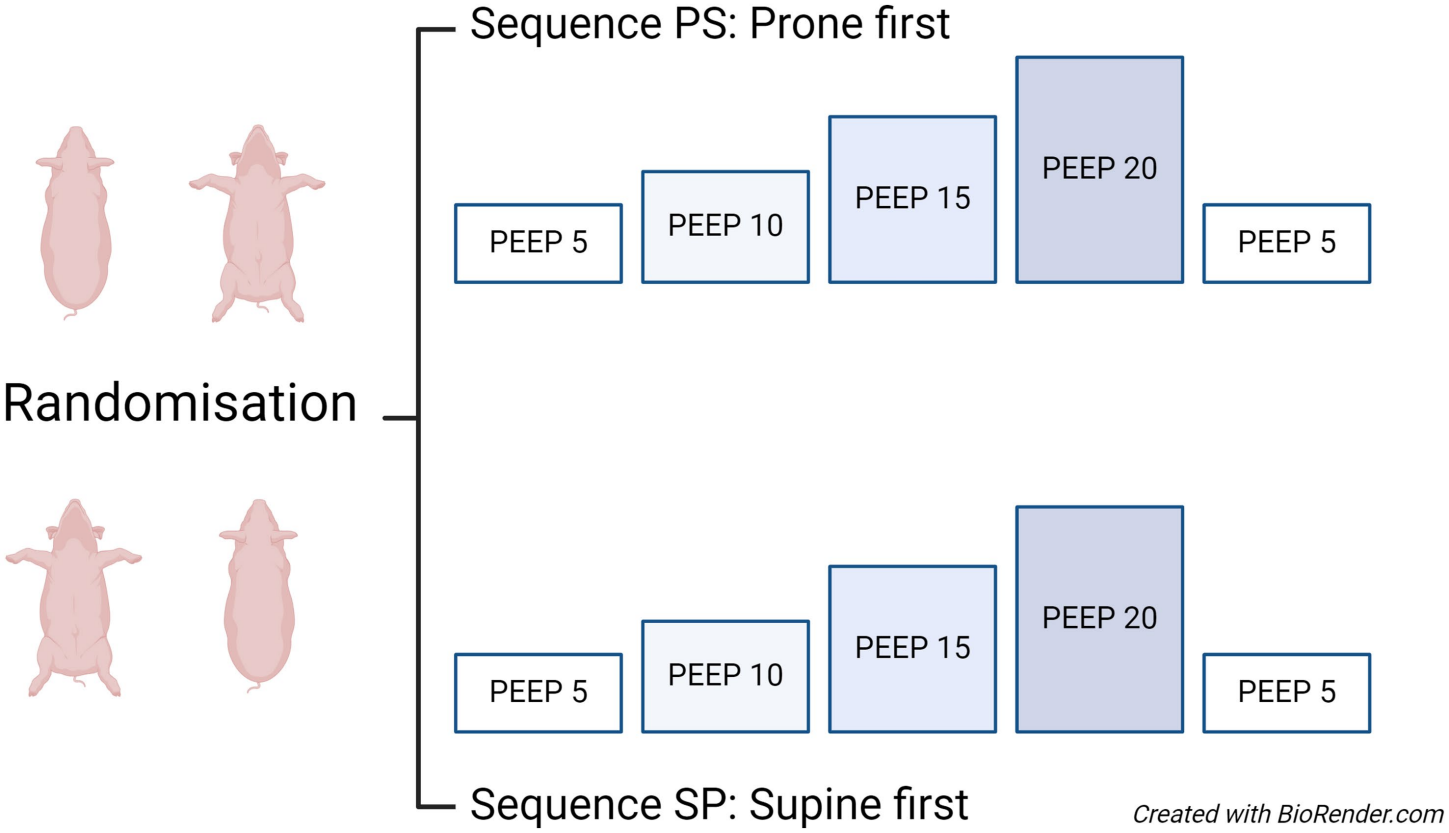
Study design: crossover design and intervention settings. The randomization scheme is shown to the left, and the periods with PEEP levels are shown to the right. Subsequent measurements were conducted at PEEP levels of 10, 15, and 20 cmH₂O, aiming to ventilate the pigs with a tidal volume of 7–8 mL/kg body weight during the PEEP trial. The prone-supine group was first exposed to the PEEP trial in the prone position, followed by the PEEP trial in the supine position. Conversely, the supine-prone group underwent the same interventions, first in the supine position and then turning to the prone position. A washout period with baseline ventilator settings was used to reduce the risk of a carry-over effect from the intervention. PEEP, positive end-expiratory pressure; PS, prone supine; SP, supine prone.

Details of the study design are illustrated in Figure 2.

Personnel not involved in the experimental procedures conducted the randomization prior to the pigs’ arrival at the laboratory. The randomization, performed by drawing lots, determined whether each pig would start in the prone or the supine position. According to this plan, the pigs were initially placed in their assigned positions at zero degrees inclination.

Systematic measurements were conducted at increasing PEEP levels of 5 (baseline), 10, 15, and 20 cmH₂O, then back to 5 cmH₂O, where PEEP 5 cmH_2_O is the baseline. Each PEEP level was maintained for 20 min. Subsequently, the pigs were repositioned to the alternate posture as per the randomization plan and the PEEP trial performed again. The ventilator settings used during the PEEP trial were volume control mode, VT of 7–8 mL/kg body weight, FiO₂ of 0.40, inspiratory: expiratory ratio of 1:2, and an inspiratory pause of 20% of the inspiratory time., and the mean values from these 20 min of measurements were used in the analysis. Additionally, respiratory mechanics was assessed by end-inspiratory and end-expiratory holds towards the end of the measurement period to ensure stable values, and followed by thermodilution measurements. Subsequently, the pigs were repositioned to the opposing posture as per the randomization plan.

### 2.5 Outcome measures

The primary outcomes were ICP and PRx. We investigated the effect of PEEP level increase on mean ICP and mean PRx and looked at the effect of position on the relationship between PEEP and ICP and Prx.

The secondary outcome was respiratory, cerebral, and hemodynamic variables related to the change in mean ICP, including baseline ICP, RAP, PaO_2_, PaCO_2_, VT, respiratory rate (RR), end-inspiratory airway pressure (Paw_ei_), elastance of the respiratory system (E_rs_), end-expiratory transpulmonary pressure (TPP_ee_), TPP_ei_, lung elastance (E_l_), mechanical power of the respiratory system (MP_rs_), CVP, PAP and CO.

### 2.6 Data processing

ICM+ software was used for data preprocessing of the high-resolution recordings before statistical analysis. Two researchers (RH and SF) scrutinized each recording visually and assessed the quality of the signals. The data were first curated manually for significant artifacts. Further, automated artifact markup was applied in ICM+. ICP values below −10 mmHg or above 60 mmHg were rejected, as were the cerebral perfusion pressure (CPP) values below 0 mmHg or above 150 mmHg. All recorded signals were downsampled to 0.1 Hz by coarse graining using 10 s, non-overlapping averages.

The PRx index was calculated using a 5-min moving window of Pearson correlation coefficients between 30 consecutive 10-s averages of ABP and ICP, updated every minute. Similarly, the compensatory reserve index (RAP) was calculated using a 5-min moving window of Pearson correlation coefficients between 30 consecutive 10-s averages of intracranial pulse pressure amplitude (AMP) and ICP, also updated every minute (18). Fisher transformation was applied to PRx and RAP before further analysis. We exported descriptive statistics values of each variable for each animal for each study period, using the event tool implementation in ICM+. Mean values were considered for all variables for further analysis.

### 2.7 Respiratory mechanics variables

Data from the ventilator was extracted from ICM+ software as one value per period.

The following variables were retrieved as absolute values:

End-inspiratory (Paw_ei_) and total end-expiratory airway pressure (PEEPtot), end-inspiratory and end-expiratory esophageal pressures (Pes_ei_ and Pes_ee_ respectively) were recorded at zero flow after a 3 s-end-inspiratory and a 3-s end-expiratory hold. Peak pressure of the respiratory system (Ppeak_rs_) and chest wall (Ppeak_cw_) were the maximal values of Paw and Pes, respectively. Respiratory rate (RR) and VT were also collected.

The following variables were calculated:

1. Peak pressure across the lung (Ppeak_l_) = Ppeak_rs_ − Ppeak_cw_.
2. Respiratory system (E_rs_), chest wall’s (E_cw_), and lung’s (E_l_) elastance was calculated according to the classic formula: E_rs_ = (Paw_ei_ − PEEPtot)/VT, E_cw_ = Pes_ei_ − Pes_ee_/VT, and E_l_ = E_rs_ − E_cw_.
3. Absolute end-expiratory TPP (TPP_ee_) was computed by subtracting Pes_ee_ to PEEPtot. Absolute end-inspiratory TPP_ei_ is calculated was calculated as the difference between Paw_ei_ and Pes_ei_. It was suggested that absolute TPP_ei_ reflect lung stress in the most dependent part of the lungs (19). Lung stress was therefore also computed from the TPP elastance (TPP_elast_) method TPP_elast_ = Paw_ei_ × (E_l_/E_rs_). TPP_elast_ was thought of as reflecting the lung stress in the non-dependent parts of the lungs.
4. MP_rs_ in J/min was calculated with the formula: MPrs = 0.098*VT*RR*(Ppeakrs − (Paw_ei_ − Paw_ee_)/2). Mechanical power of the dependent lung (MPlDep) = 0.098*VT*RR*(Ppeakl − (TPP_ei_ − TPP_ee_)/2). Mechanical power of the non-dependent lung (MPlnonDep) = 0.098*VT*RR*(Ppeaklung − (TPP_elast_ − TPP_ee_)/2).

### 2.8 Statistical analysis

The following complete set of physiological variables were considered in the analysis:

1. Cerebral and hemodynamic variables: ICP, PRx, RAP, AMP, ABP, CPP, CVP, PAP, CO, temperature.
2. Gas exchange and respiratory variables: SpO_2_, PaCO_2_, PaO_2_, pH, PaO_2_/FiO_2_-ratio, RR, Paw_ei_, Pes_ei_, Pes_ee_, TPP_ei_, TPP_ee_, TPP_elast_, E_rs_, E_cw_, E_l_, MP_rs_, MP_lDep_, MP_lnonDep_, EVLW, SVV, PEEP, VT, Ppeak_rs_, Ppeak_cw_, and Ppeak_l_.

The normality of continuous variables was assessed with the Shapiro–Wilks test. The homogeneity of variance was also evaluated. Continuous variables were presented as mean and standard deviation (SD) or median and interquartile range (IQR). Outlier analysis was conducted using graphical methods, revealing no outliers in the data after scrutinization during the data preprocessing.

### 2.9 Statistical analysis strategy

The assumptions of the crossover design (absence of carry-over effect, absence of sequence (prone or supine first) effect, and period of intervention) were tested for change in ICP and PRx with linear mixed effect models using type of intervention (PEEP increase), sequence, and period of intervention (first or second) as a fixed effect, with interaction terms, and the animal and position as random effect variables. Furthermore, the absence of a carry-over effect was tested with a paired *t*-test (two-tailed) on baseline or washout periods for ICP and PRx.

#### 2.9.1 Primary endpoints

We examined whether different levels of PEEP influence ICP and PRx in prone and supine position.

- The difference between baseline variables in prone and supine positions was tested using two-tailed *t*-test or the Mann–Whitney test, depending on the data distribution.
- The treatment effects were evaluated by comparing values during the intervention periods (PEEP 10, 15, 20 cmH_2_O) to the baseline period (PEEP 5 cmH_2_O).
- After checking for assumptions, two-way ANOVA was performed to see if there was a significant change in ICP between prone and supine across the different PEEP levels.
- If the linear mixed-effects model assumption confirmed that the response in mean ICP and PRx to the intervention (different PEEP levels) did not differ between the positions, the mean changes from baseline to intervention across supine and prone positions were pooled and analyzed through a repeated measure one-way ANOVA.

#### 2.9.2 Secondary endpoints

Using scatterplots and linear mixed models, we investigated the relationships between the changes in ICP induced by the intervention and various baseline neurological, hemodynamic, and respiratory variables. For this analysis, the prone and supine data were pooled since the baseline variables apart from ICP and RAP, were not different between the groups. The covariables were empirically chosen based on clinical relevance to the mechanisms by which PEEP might affect ICP, and not all recorded variables were explored.

We tested the following baseline variables: ICP, RAP, PaO_2_, PaCO_2_, VT, RR, Paw_ei_, TPP_ei_, TPP_ee_, E_rs_, E_l_, MP_rs_, CVP, PAP, CO.

ICP was included due to prior evidence suggesting that patients with higher baseline ICP may exhibit a different response to PEEP (20). RAP, as a crude index of brain compliance, was considered relevant as reduced compliance might amplify ICP changes in response to increased PEEP.

PaO₂ and PaCO₂ were included due to their impact on cerebral oxygenation and vasoreactivity, with PaCO₂ being a potent regulator of cerebral blood flow, which in turn affects ICP. Paw_ei_, TPP_ei_, TPP_ee_, E_rs_, E_l_ were included because they may affect how thoracic pressures are transmitted to the brain. Similarly, CVP and PAP were examined due to their role in venous return and pulmonary hemodynamics, which can influence cerebral venous drainage and ICP. MP_rs_, VT and RR was included to evaluate the total mechanical energy transmitted to the lungs, which could impact PEEP’s effects on the brain. Lastly, CO was considered due to its potential influence on cerebral blood flow, as PEEP may reduce venous return and cardiac output. A *p*-value <0.05 was considered statistically significant. Since we only analyzed clinically relevant variables and did not examine all recorded variables, multiple comparison corrections were not applied.

The statistical analysis was performed with R software version 4.3 (21).

## 3 Results

### 3.1 Baseline characteristics

Table 1 presents variables measured during baseline PEEP 5 cmH_2_0 in prone or supine positions before the stepwise increase in PEEP. Mean ICP at baseline was higher in prone (P5) than supine (S5) (*p* < 0.01). RAP differed at baseline between prone and supine (*p* < 0.01). No other variables were different between prone and supine before randomization.

**Table 1:** Baseline physiological values for study animals.

Variable	All animals (*n* = 12)		
	P5 (*n* = 8)	S5 (*n* = 4)	*p*
Cerebral variables			
ICP (mmHg)	6 ± 4	12 ± 3	0.03
PRx	−0.129 ± 0.238	−0.109 ± 0.105	0.87
RAP	−0.090 ± 0.153	0.192 ± 0.105	0.01
CPP (mmHg)	78 ± 13	69 ± 5	0.25
Respiratory variables			
PaCO_2_ (mmHg)	43 ± 4	41 ± 4	0.42
PaO_2_ (mmHg)	188 ± 30	188 ± 15	0.94
PaO_2_/FiO_2_ (mmHg)	470 ± 75	470 ± 38	1.00
VT (mL)	179 (174–183)	179 (174–193)	0.86
RR (breaths/min)	31 ± 7	28 ± 2	0.37
Ppeak_rs_ (cmH_2_O)	20.5 ± 4.4	17.7 ± 0.5	0.12
Ppeak_cw_ (cmH_2_O)	8.8 ± 3.9	9.4 ± 2	0.79
Ppeak_l_ (cmH_2_O)	11.6 ± 3.9	8.3 ± 2.1	0.14
Paw_ei_ (cmH_2_O)	14.2 ± 3.0	12.9 ± 0.6	0.27
Paw_ee_ (cmH_2_O)	4.5 ± 0.5	4.5 ± 0.2	0.91
TPP_ei_ (cmH_2_O)	6.9 ± 3.2	4.6 ± 1.9	0.22
TPP_ee_ (cmH_2_O)	0.5 ± 2.1	−1 ± 1.9	0.24
TPP_elast_ (cmH_2_O)	9.2 ± 3.7	8.7 ± 2.2	0.80
Pes_ei_ (cmH_2_O)	7.3 ± 3.3	8.3 ± 1.6	0.58
Pes_ee_ (cmH_2_O)	4 ± 2.1	5.5 ± 1.6	0.24
E_rs_ (cmH_2_O/l)	53 (47–59)	47 (42–50)	0.29
E_cw_ (cmH_2_O/L)	19 (14–23)	15 (9–21)	0.55
E_l_ (cmH_2_O/L)	33 (24–41)	26 (26–31)	0.81
MP_rs_ (J/min)	7 (6–11)	7 (6–7)	0.68
MP_lDep_ (J/min)	5 (3–6)	2.8 (2–3)	0.10
MP_lnonDep_ (J/min)	5 (3–6)	3 (2–3)	0.09
Hemodynamic variables			
ABP (mmHg)	80 (75–90)	80 (80–82)	0.93
CVP (mmHg)	12 ± 2	13 ± 2	0.20
PAP (mmHg)	20 ± 4	22 ± 4	0.46
CO (L/min)	3.6 ± 1	4.3 ± 0.7	0.28
EVLW (mL)	267 (244–366)	277 (253–312)	0.68
SVV (%)	6 ± 3	8 ± 3	0.30
Body temperature (°C)	38.5 (38.2–38.5)	38.5 (38.4–38.5)	0.77

The table presents baseline physiological values measured before intervention and *p*-values for group comparison. Values are presented as mean ± SD or median (IQR). *p*: *p*-values comparing the prone and supine groups (two-sample *t*-test for normally distributed variables and Mann–Whitney *U* test for non-normally distributed variables). P5: Animals starting interventions in prone, positive end-expiratory pressure 5 cmH_2_O. S5: Animals starting interventions in supine, positive end-expiratory pressure 5 cmH_2_O. E_rs_, E_cw_, MP_lDep_, and MP_lnonDep_ were normally distributed but presented as median and IQR to allow comparison with non-normally distributed variables E_l_ and MP_rs_.

Tables with respiratory and hemodynamic variables at different PEEP levels in supine and prone are presented in the Supplementary Tables 2A,B.

### 3.2 Effects of PEEP on ICP and PRx

**Figure 3:**
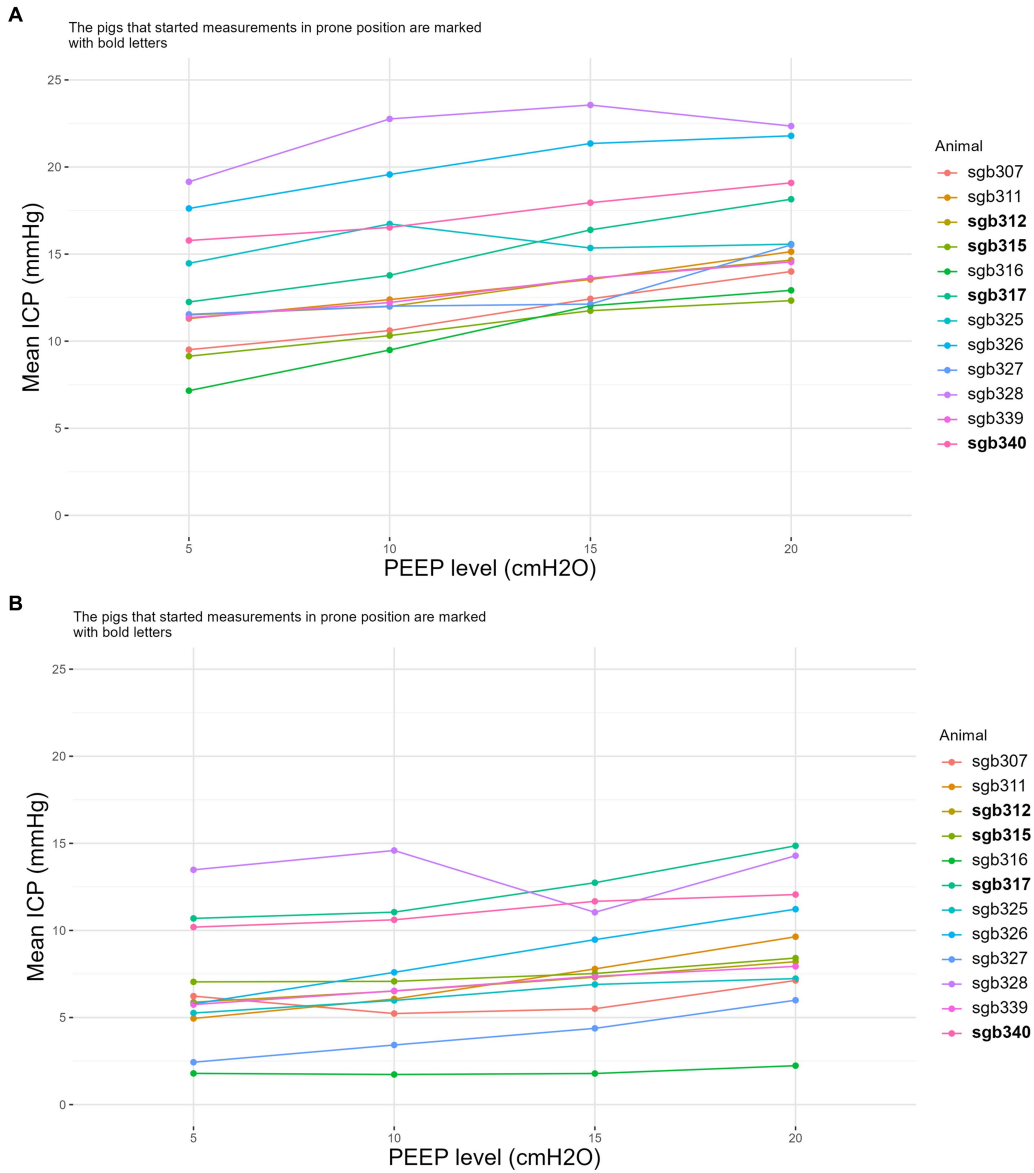
The effect of PEEP and animal position on ICP. Mean intracranial pressure (ICP) in the supine position **(A)** and in the prone position **(B)** with PEEP at 5, 10, 15 and 20 cmH_2_O. Each line represents an individual animal, identified by their unique identifiers (e.g., sgb307, sgb311). The identifiers in bold are the animals that started supine first. ICP, intracranial pressure; PEEP, positive end-expiratory pressure.

Figure 3 shows each animal’s mean ICP during each PEEP level, in prone and supine.

The study design assumptions were fulfilled for the analysis of the primary outcome. Change in ICP and PRx had no carry-over, sequence, or period effect.

**Figure 4:**
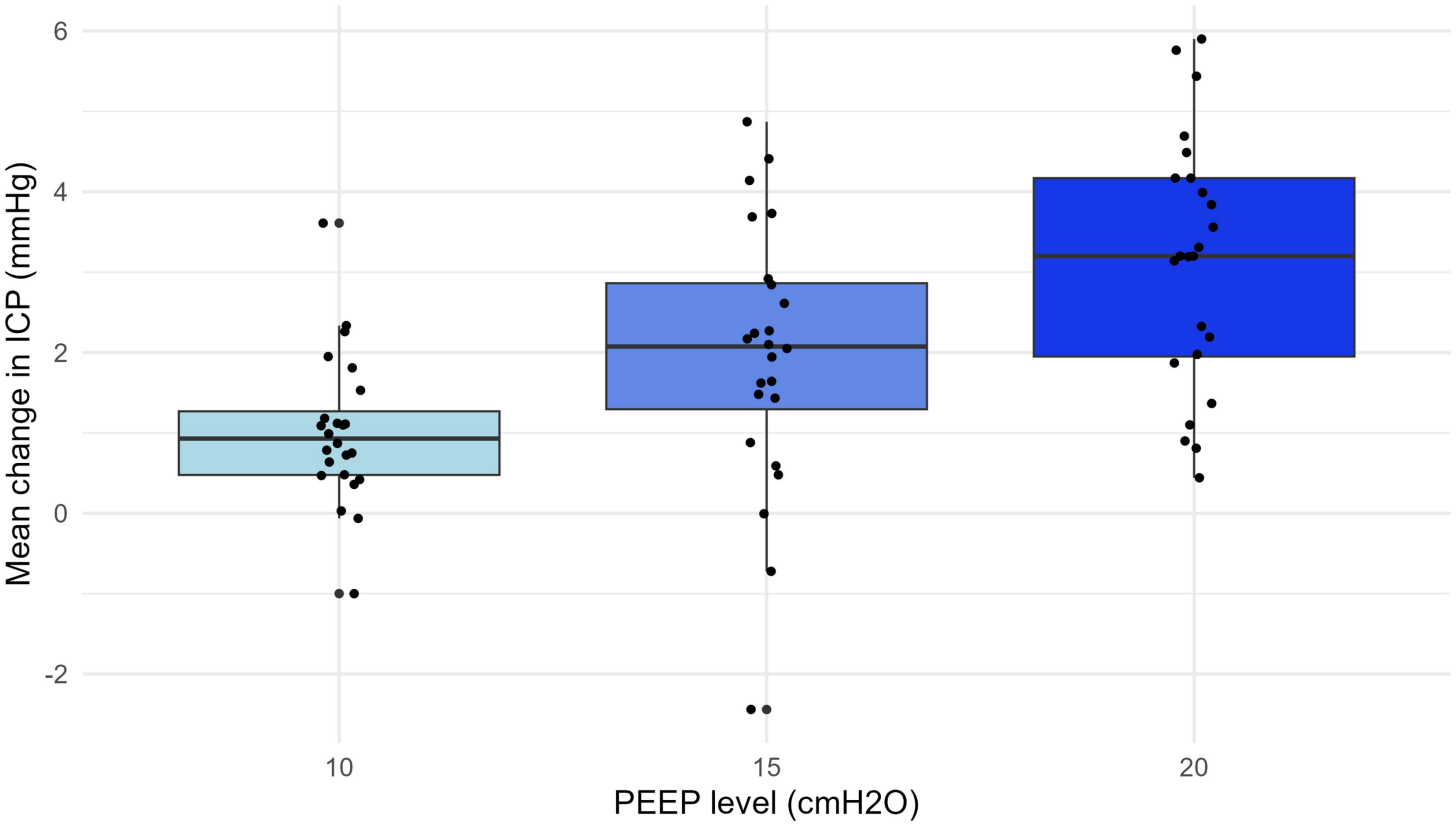
Mean change in ICP with PEEP at 10, 15, and 20 cmH_2_O. The change in intracranial pressure (ICP) was calculated as the increase from the baseline at positive end-expiratory pressure (PEEP) 5 cmH_2_O. There was a significant association between PEEP levels and change in mean ICP (*r* = 0.25, *p* < 0.001). The prone and supine data are pooled. ICP, intracranial pressure; PEEP, positive end-expiratory pressure.

There was a significant association between PEEP levels and change in mean ICP (*p* < 0.001) (Figure 4, supine and prone are pooled). Two-way ANOVA showed no interaction between PEEP levels and position (*p* = 0.66), i.e., the effect of increased PEEP on the absolute rise of ICP was not influenced by position. Position affected change in ICP, regardless of PEEP (*p* < 0.001) (Supplementary Figures A1, A2). The average ICP changes observed at PEEP settings of 10, 15, and 20 cmH_2_O, compared to a baseline of 5 cmH_2_O, were 0.6 mmHg ± 3.5, 1.2 mmHg ± 3.1 in prone, and 2.5 mmHg ± 3.6, and 1.5 mmHg ± 4.1, 2.7 mmHg ± 3.9, and 3.8 mmHg ± 3.3, in supine*.*

**Figure 5:**
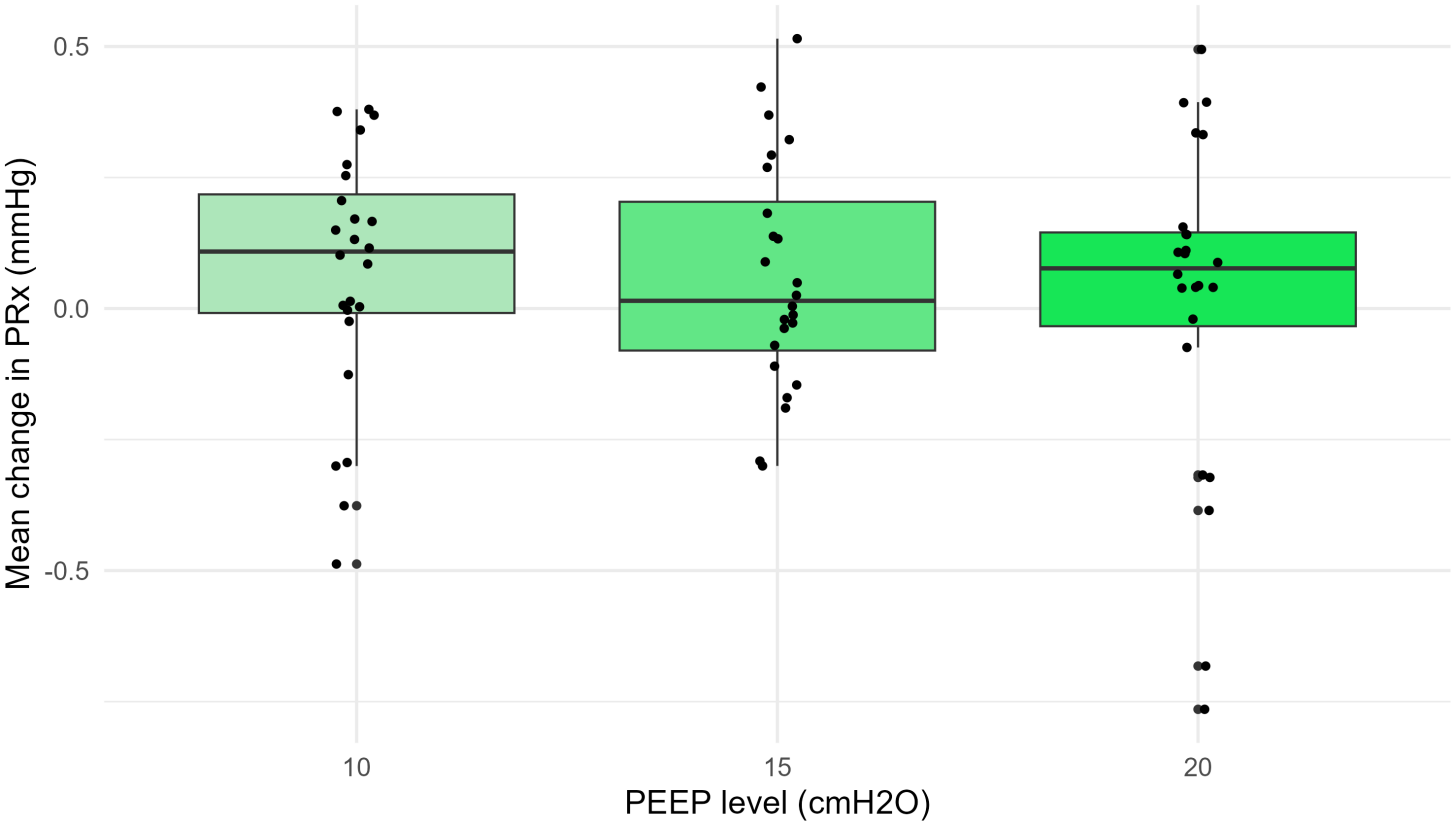
Mean change in PRx with PEEP at 10, 15, and 20 cmH_2_O. The increase in PEEP did not significantly change the mean pressure reactivity index (PRx) compared to the baseline values (*p* = 0.7). ICP, intracranial pressure; PEEP, positive end-expiratory pressure; PRx, pressure reactivity index.

The PEEP increase did not significantly change mean PRx compared to the baseline value (Figure 5, supine and prone are pooled). The average PRx changes observed at PEEP settings of 10, 15, and 20 cm H_2_O, compared to a baseline of 5 cmH_2_O, were 0.1 ± 0.16, 0.0 ± 0.17, and − 0.1 ± 0.28 in prone, and 0.1 ± 0.24, 0.1 ± 0.21, and 0.1 ± 0.24 in supine (Supplementary Figure A3).

### 3.3 Exploratory analysis

Exploratory analysis looked at baseline variables that could be correlated to changes in ICP. We found that PEEP-induced increases in ICP was higher in cases of lower E_rs_ (*p* = 0.024, *r* = −0.21), lower TPP_ee_ (*p* = 0.009, *r* = −0.20), and lower TPP_ei_ (*p* = 0.006, *r* = −0.34). In addition, higher CVP (*p* = 0.0032, *r* = 0.30) and higher baseline ICP (*p* = 0.012, *r* = 0.20) were associated with greater changes in ICP.

RAP, PaO_2_, PaCO_2_, VT, RR, Paw_ei_, E_l_, MP_rs_, PAP, and CO were not significantly correlated with ICP. The correlation plots for the significant correlations are presented in Supplementary Figures B1–B5.

## 4 Discussion

Our main findings in this experimental porcine model are: (1) increased PEEP levels significantly increase ICP, regardless of position. (2) Both PEEP and position independently affect ICP. (3) PRx remains unaffected by changes in PEEP in supine and prone positions. (4) Baseline ICP, CVP, TPP_ei_, TPP_ee_ and E_rs_ are correlated with an elevated ICP response.

### 4.1 Effect of PEEP on ICP

Our study shows a relatively modest rise in ICP, even with PEEP settings as high as 20 cmH_2_O. An increase in ICP by 1 mmHg with a 5 cmH_2_O increase in PEEP appears clinically insignificant.

Other animal studies confirm increases in ICP with elevated PEEP levels. Huseby et al. (22) examined dogs with healthy lungs and normal ICP and reported a mean increase in ICP in dogs of 10 mmHg when PEEP was increased from 0 to 20 cmH₂O. While PaCO₂ was maintained at stable levels in this study, blood pressure was generally allowed to fluctuate without intervention which might have influenced the observed changes in ICP that are greater than ours. Aidinis et al. (23) observed a small, clinically insignificant increase in ICP in cats with intracranial hypertension and healthy lungs when increasing PEEP to 15 cmH₂O.

Sun et al. (24) demonstrated that elevated PEEP increased ICP in dogs with healthy lungs and normal ICP. The mean increase was, similar to our findings, only 5 mmHg with a PEEP increase from 0 to 20 cmH_2_O. A recent systematic review on the effect of PEEP on ICP (25) states that PEEP can increase ICP, but the extent of this increase varies across different studies and patient populations. Beqiri et al. (20) found that while most patients with ABI and healthy lungs showed a significant but clinically non-relevant increase in ICP, nearly one-quarter of the patients experienced a rise in ICP during the intervention to above 20 mmHg, necessitating its interruption. The effect of PEEP on ICP have also been tested perioperatively during neurosurgery. Ruggieri et al. (26) found no difference in ICP when applying LPV (7 mL/kg and PEEP 5 cmH₂O) and traditional ventilation (9 mL/kg and PEEP 0 cmH₂O) in patients undergoing surgery for brain tumour. Both animal and human studies align with our results, indicating that PEEP can increase ICP. However, the clinical significance of these increases varies widely. This variability underscores the need for careful, individualized management of increasing PEEP.

### 4.2 Effect of prone versus supine position on ICP

The baseline ICP and RAP index were significantly higher in the supine position compared to the prone position, suggesting slightly reduced cerebral compliance in the supine posture. In humans, ICP is also dependent on position. While human studies typically show higher ICP in the prone position (27, 28), our findings in pigs indicate the opposite, likely due to anatomical differences between species. In pigs, the brain is smaller and positioned more ventrally and caudally, influencing ICP measurements based on the head’s orientation. Specifically, in the prone position, the frontal cerebral parenchyma is located anteriorly, whereas in the supine position, it shifts posteriorly. The placement of the ICP probe in the frontal cerebral parenchyma likely results in increased gravitational pressure when turning from prone to supine, resulting in higher ICP readings. The positional dependence of ICP has been confirmed by other porcine studies (29, 30), though exploring these mechanisms was beyond the scope of our study.

Baseline ICP measurements in the supine position vary across animal studies. For example, studies with healthy pigs have reported ICP values consistent with our findings (29, 31) while others report lower values (32). Our study’s baseline ICP in the prone position is consistent with findings from a study in healthy pigs (33), but to our knowledge, no other studies have compared ICP between prone and supine positions within the same animal.

We found that the absolute mean changes in ICP were significantly greater in the supine position, regardless of PEEP levels, likely due to the higher baseline ICP in this posture. Several human studies indicate increased ICP in the prone position compared to the supine position, independent of PEEP level (28, 34). However, Thelandersson et al. (35) found no increase in ICP in the prone position in patients with reduced intracranial compliance, suggesting variability based on specific patient conditions.

### 4.3 Effect of PEEP on PRx

While the offset of ICP increased following the increase in PEEP, as indicated by the rise in the mean ICP value, the cerebral arterioles maintained their ability to constrict and dilate in response to changes in perfusion pressure, as reflected by the unchanged PRx. Previous animal studies on PRx have used hyper-/hypotension or ABP oscillations to investigate and validate the use of PRx (16, 36–38). To our knowledge, our study is the first to explore the relationships between PEEP increase and intracranial waveform analysis/autoregulation in a porcine model.

Two recent human studies showed no worsening of PRx with PEEP increase (20, 39). This aligns with our findings. Our findings indicate that PEEP adjustments do not significantly impact PRx, suggesting that cerebrovascular autoregulation remains stable under varying PEEP conditions. This stability implies that cerebral autoregulation mechanisms can withstand ventilatory changes without compromising cerebrovascular integrity.

A recent review (40) highlights the importance of PRx in managing traumatic brain injury, emphasizing how intracranial waveform analysis can improve patient outcomes. Given the scarcity of studies examining PRx in healthy brains, our research holds clinical significance. Understanding the response of a healthy brain to increased PEEP can provide valuable insights into the mechanisms affecting the brain in various pathological conditions where cerebral autoregulation is critical.

### 4.4 Baseline variables correlated to change in ICP

Our study found a significant positive correlation between changes in mean ICP induced by PEEP and position with baseline values of ICP and CVP, and a negative correlation with E_rs_ and TPP_ei_ and TPP_ee_.

The positive correlation between baseline ICP and the ICP response to PEEP changes confirms findings from our group’s previous study conducted on ABI patients (20). However, unlike in the clinical study, the RAP index did not differ between baseline and intervention phases, leaving the role of brain compliance uncertain. These results suggest that patients with elevated baseline ICP are at a higher risk of significant ICP increases when PEEP is adjusted. Therefore, close monitoring and cautious titration of PEEP are warranted.

Higher baseline CVP was associated with increased ICP, possibly due to reduced cerebral venous drainage. Chen et al. (41) also reported a correlation between CVP and increased ICP in animals with normal ICP, though they did not explore the correlation with baseline CVP. This finding suggests that in vulnerable patients, where avoiding ICP increases is critical, monitoring CVP during PEEP adjustments could be beneficial to identify those at higher risk of impaired cerebral venous outflow and subsequent ICP elevation.

Our study observed that the effect of PEEP on ICP was smaller in animals with higher baseline E_rs_. Contrarily, Chen et al. reported increased ICP in response to PEEP in an experimental pig study when elastance was increased with chest wall strapping. The E_rs_ with chest wall strapping was much lower than in normal lungs, which might explain the difference from our study. They did not report the correlation between ICP increase and baseline E_rs_ in normal lungs. In an observation study on patients, Robba et al. (42) found no correlation between baseline respiratory system compliance and ICP increase with PEEP. Similarly, a clinical intervention study did not find associations between baseline respiratory compliance and the responses of ICP to PEEP (20). Our finding suggests that patients with reduced respiratory compliance may tolerate higher levels of PEEP without significant increases in ICP. However, as previous studies have reported conflicting results, further research is needed to clarify the interaction between respiratory mechanics and cerebral hemodynamics.

Our results suggest that higher baseline TPP_ei_ and TPP_ee_ predicts a lower ICP response to PEEP. In this study, higher baseline TPP_ei_ and TPP_ee_ was attributed to variations in intrathoracic pressure (measured as Peso) or compliance, as PEEP and volume were set. To our knowledge, only one study reports the role of transpulmonary pressures on ICP (20). Baseline TPP_ei_ and TPP_ee_ in patients with ABI but no lung injury did not predict a prominent ICP increase.

### 4.5 Study strengths and limitations

Our study’s strengths include the use of a controlled experimental model, which allows for precise manipulation of variables and thorough monitoring. This is the first study with multimodal respiratory, hemodynamic and cerebral monitoring that examines the effects of PEEP in both prone and supine positions, providing baseline data for future studies involving pathological models.

Importantly, our study examined the physiological responses to PEEP in animals without ABI, and thus our findings may not reflect the responses in mechanically ventilated ABI patients. However, our findings provide much-needed baseline data for future studies in animals with raised ICP, disturbed cerebrovascular autoregulation, and/or damaged lungs.

Limitations include the use of an animal model, which may not fully replicate human physiology. The anatomical differences might result in baseline ICP values that may not correspond to humans in different positions. The small sample size limits the generalizability of our findings. The skewed randomization may also affect the reliability of our results. Animals were positioned at zero degrees without head elevation, which differs from typical clinical practice in humans. We wanted to minimize the risk of a PEEP effect on MAP, to have the most controlled settings possible for evaluating isolated ICP and PRx responses. Also in clinical practice, head elevation is reduced if it affects MAP and there is no ICP problem. Furthermore, PEEP levels were not randomized, and all animals were exposed to increasing PEEP in the same order, which could influence the results. We chose this approach because the main aim was to examine the ICP and PRx response with incremental PEEP in the prone and supine positions. This approach reflects typical clinical practice in which PEEP is gradually increased to monitor response.

As lung recruitment maneuvers were not performed between PEEP levels, this may potentially have affected the consistency of lung mechanics measurements. We chose not to perform a procedure that could affect ICP and PRx and would require extending the study with a longer time for stability between PEEP levels. Additionally, the short duration of the experiments did not allow for the assessment of the long-term effects of PEEP. A limitation is also that we did not have other cerebral monitoring than ICP.

## 5 Conclusion

To our knowledge, this study is the first on ICP changes in pigs comparing PEEP increase in prone and supine positions. Our findings suggest that increases in PEEP elevate ICP slightly, independently of position, without negatively impacting cerebral autoregulation (CA) in pigs with normal ICP and healthy lungs. We identified that the baseline variables ICP, CVP, E_rs_, TPP_ei_ and TPP_ee_ might predict elevations in ICP, but further investigation is warranted, especially targeting this issue as a primary objective.

Further exploration of the PEEP-ICP-CA relationship, particularly in more complex conditions such as ARDS and intracranial hypertension, is essential. This study provides a basis for such investigations.

## Data Availability

The raw data supporting the conclusions of this article will be made available by the authors, without undue reservation.

## Glossary

ABP: Arterial blood pressure
CO: Cardiac output
CPP: Cerebral perfusion pressure
CVP: Central venous pressure
E_cw_: Elastance of the chest wall
E_l_: Lung elastance
E_rs_: Elastance of the respiratory system
EVLW: Extravascular lung water
ICP: Intracranial pressure
MP_lDep_: Mechanical power of the dependent lung
MP_lnonDep_: Mechanical power of the non-dependent lung
MP_rs_: Mechanical power of the respiratory system
PaCO_2_: Arterial partial pressure of carbon dioxide
PaO_2_: Arterial partial pressure of oxygen
PaO_2_/FiO_2_: Ratio of PaO_2_ to inspired fraction of oxygen in air
Paw_ee_: End-expiratory airway pressure
Paw_ei_: End-inspiratory airway pressure
PAP: Pulmonary artery pressure
Pes_ee_: End-expiratory esophageal pressure
Pes_ei_: End-inspiratory esophageal pressure
Ppeak_cw_: Peak inspiratory pressure of the chest wall
Ppeakl - Lung peak pressure
Ppeak_rs_: Peak inspiratory pressure of the respiratory system
PRx: Pressure reactivity index
RAP: Compensatory reserve index
RR: Respiratory rate
SVV: Stroke volume variation
TPP_ei_: End-inspiratory transpulmonary pressure
TPP_ee_: End-expiratory transpulmonary pressure
TPP_elast_: Elastance-derived transpulmonary pressure
VT: Tidal volume
