# Supplementary material for: Positive end-expiratory pressure increases intracranial pressure but not pressure reactivity index in supine and prone positions: a porcine model study
Source: Front Med (Lausanne). 2025 Jan 7;11:1501284. doi: 10.3389/fmed.2024.1501284 (PMC11747722; doi:10.3389/fmed.2024.1501284)
Supplement: Supplementary file 1 [file Data_Sheet_1.pdf]

Supplementals

Supplementary Table 2A

Respiratory and hemodynamic variables at different PEEP-levels in supine

**Respiratory and hemodynamic variables at different PEEP-levels in supine**					
Variable	S5	S10	S15	S20	p
Respiratory Variables					
$PaCO_2$ (mmHg)	41 (38-45)	42 (38-47)	43 (39-47)	44 (41-47)	0.35
PaO_2 (mmHg)	180 (150-195)	180 (143-195)	188 (150-203)	188 (150-210)	0.68
PaO_2/FiO_2 (mmHg)	450 (375-488)	450 (358-488)	470 (375-508)	470 (375-525)	0.68
VT_{insp} (ml)	183 (172-183)	183 (174-183)	183 (174-183)	174 (154-185)	0.82
RR (breaths/min)	28 (27-31)	28 (27-31)	30 (28-36)	38 (34-40)	0.02
$Ppeak_{rs}$ (cmH_2O)	17.4 (16.9-19.2)	24.1 (22.7-28.6)	29.6 (28.4-32.2)	37.8 (36.7-40.1)	<0.01
$Ppeak_{cw}$ (cmH_2O)	9.8 ± 1.6	11.5 ± 2.5	13.1 ± 2.2	15.4 ± 4.0	<0.01
$Ppeak_{l}$ (cmH_2O)	8.8 ± 3.4	13.7 ± 3.2	17.6 ± 4.2	23.8 ± 6.5	<0.01
Paw_{ei} (cmH_2O)	12.5 (12.1-13.6)	18.9 (18.0-19.8)	24.9 (24.5-25.6)	31.7 (31.0-32.5)	<0.01
Paw_{ee} (cmH_2O)	4.6 (4.4-4.7)	9.9 (9.8-10.1)	15.1 (15.0-15.2)	20.4 (20.2-21.0)	<0.01
TPP_{ei} (cmH_2O)	4.6 (4.2-4.8)	9.4 (8.9-9.8)	13.5 (13.1-14.2)	18.4 (17.2-20.9)	<0.01
TPP_{ee} (cmH_2O)	-0.9 ± 1.2	2.9 ± 0.8	6.1 ± 1.2	9.8 ± 2.1	<0.01
TPP_{elast} (cmH_2O)	8.6 ± 2.5	14.3 ± 3.0	20.1 ± 3.6	25.3 ± 5.5	<0.01
Pes_{ei} (cmH_2O)	8.1 ± 1.2	9.4 ± 1.1	11.3 ± 1.6	13.3 ± 3.4	<0.01
Pes_{ee} (cmH_2O)	5.6 ± 1.1	7.2 ± 0.7	9.1 ± 1.3	10.9 ± 2.5	<0.01
E_{rs} (cmH_2O/l)	44 ± 7	49 ± 8	57 ± 9	67 ± 14	<0.01
E_{cw} (cmH_2O/l)	14 ± 7	12 ± 5	14 ± 6	15 ± 7	0.78
E_{l} (cmH_2O/l)	29 ± 8	37 ± 9	44 ± 8	52 ± 13	<0.01
MP_{rs} (J/min)	6(6-10)	9 (9-12)	13 (12-19)	20 (17-25)	<0.01
MP_{lDep} (J/min)	3 (2-4)	5 (4-6)	8 (6-11)	12 (9-16)	<0.01
$MP_{lnonDep}$ (J/min)	2 (1-4)	4 (3-5)	6 (5-9)	10 (8-14)	<0.01
Hemodynamic Variables					
ABP (mmHg)	81 ± 4	82 ± 6	87 ± 5	83 ± 6	0.05
CVP (mmHg)	14 ± 2	15 ± 2	17 ± 3	17 ± 3	<0.01
PAP (mmHg)	22 (18-25)	25 (23-27)	29 (28-32)	34 (30-35)	<0.01
CO (L/min)	3.4 ± 0.9	3.4 ± 0.8	3.4 ± 0.8	3.4 ± 0.8	0.99
EWLW (mL)	297 (252-316)	Not measured	Not measured	283 (251-310)	0.69
SVV (%)	7 ± 2	Not measured	Not measured	15 ± 6	<0.01
Temperature (°C)	38.4 ± 0.2	38.4 ± 0.2	38.4 ± 0.4	38.4 ± 0.3	0.95

Supplementary Table 2B

Respiratory and hemodynamic variables at different PEEP-levels in supine

**Respiratory and hemodynamic variables at different PEEP-levels in prone**					
PEEP level	PEEP 5	PEEP 10	PEEP 15	PEEP 20	p
Respiratory Variables					
$PaCO_2$ (mmHg)	42 (41-44)	41 (40-43)	41 (39-43)	42 (41-48)	0.52
PaO_2 (mmHg)	188 (165-218)	188 (158-203)	203 (158-218)	203 (158-218)	0.83
PaO_2/FiO_2 (mmHg)	470 (413-545)	470 (395-508)	508 (395-545)	508 (395-545)	0.83
VT_{insp} (ml)	179 (174-183)	174 (165-183)	175 (172-183)	156 (142-183)	0.42
RR (breaths/min)	31 ± 6	33 ± 7	35 ± 8	39 ± 10	0.09
$Ppeak_{rs}$ (cmH_2O)	18.4 (17.5-19.6)	23.9 (21.6-25.7)	30.0 (29.1-35.8)	39.2 (37.6-44.0)	<0.01
$Ppeak_{cw}$ (cmH_2O)	7.9 ± 4.1	9.2 ± 4.4	12.8 ± 4.7	15.8 ± 4.8	<0.01
$Ppeak_{l}$ (cmH_2O)	11.5 ± 4.0	15.8 ± 4.9	19.1 ± 6.7	25.9 ± 8.4	<0.01
Paw_{ei} (cmH_2O)	12.8 (11.7-13.5)	18.1 (16.5-20.4)	24.3 (23.8-26.8)	33.1 (32.6-34.1)	<0.01
Paw_{ee} (cmH_2O)	4.4 (4.2-4.6)	9.9 (9.7-10.1)	15.2 (15.1-15.3)	20.4 (20.2-20.7)	<0.01
TPP_{ei} (cmH_2O)	7.2 ± 3.2	11.1 ± 4.5	14.8 ± 3.9	21.1 ± 6.4	<0.01
TPP_{ee} (cmH_2O)	1.0 ± 2.3	4.4 ± 2.4	7.1 ± 2.8	10.5 ± 3.1	<0.01
TPP_{elast} (cmH_2O)	8.7 (6.7-10.5)	13.6 (11.2-15.3)	18.7 (17.0-20.1)	26.0 (22.0-28.3)	<0.01
Pes_{ei} (cmH_2O)	6.2 ± 3.6	7.4 ± 3.7	10.7 ± 3.7	13.0 ± 4.3	<0.01
Pes_{ee} (cmH_2O)	3.4 ± 2.3	5.5 ± 2.6	8.2 ± 3.0	10.0 ± 3.2	<0.01
E_{rs} (cmH_2O/l)	49 (41-57)	50 (38-54)	56 (49-65)	86 (74-90)	<0.01
E_{cw} (cmH_2O/l)	16 ± 9	10 ± 10	14 ± 6	19 ± 10	0.12
E_{l} (cmH_2O/l)	33 ± 11	37 ± 15	42 ± 10	63 ± 14	<0.01
MP_{rs} (J/min)	7 (6-10)	10 (9-15)	15 (13-22)	21 (16-27)	<0.01
MP_{lDep} (J/min)	5 ± 2	7 ± 3	10 ± 5	14 ± 7	<0.01
$MP_{lnonDep}$ (J/min)	5 ± 2	7 ± 3	9 ± 5	12 ± 7	<0.01
Hemodynamic Variables					
ABP (mmHg)	82 (76-86)	79 (76-82)	80 (75-85)	80 (76-84)	0.91
CVP (mmHg)	11 ± 2	12 ± 2	13 ± 2	14 ± 3	0.01
PAP (mmHg)	21 ± 4	22 ± 4	25 ± 4	29 ± 5	<0.01
CO (L/min)	3.7 ± 0.9	3.6 ± 0.8	3.6 ± 0.8	3.6 ± 0.8	0.97
EWLW (mL)	316 ± 145	Not measured	Not measured	303 ± 52	0.79
SVV (%)	7 ± 3	Not measured	Not measured	9 ± 3	0.2
Body temperature (°C)	38.5 ± 0.2	38.5 ± 0.4	38.4 ± 0.4	38.3 ± 0.4	0.36

Table 2A and 2B The tables present respiratory and hemodynamic variables at different PEEP levels in supine (A) and prone (B). Values are presented as mean ± SD or as median (IQR).

P: p-values indicate whether there are statistically significant differences between PEEP levels for each variable (ANOVA for normally distributed variables with equal variance, Welch's ANOVA for normally distributed variables with unequal variance, and Kruskal-Wallis test for non-normally distributed variables).

<u>Abbreviations:</u>

PEEP: positive end-expiratory pressure; ABP: mean arterial blood pressure; CO: cardiac output; CVP: mean central venous pressure; E_{cw}: elastance of the chest wall; E_{l}: lung elastance; E_{rs}: elastance of the respiratory system; EVLW: extravascular lung water; MP_{lDept}: mechanical power of the dependent lung; $MP_{lnonDep}$: mechanical power of the non-dependent lung; MP_{rs}: mechanical power of the respiratory system; $PaCO_2$: arterial partial pressure of carbon dioxide; PaO_2: arterial partial pressure of oxygen; PaO_2/FiO_2: ratio of PaO_2 to inspired fraction of oxygen in air; Paw_{ee}: end-expiratory airway pressure; Paw_{ei}: end-inspiratory airway pressure;PAP: mean pulmonary artery pressure; Pes_{ee}: end-expiratory esophageal pressure; Pes_{ei}: end-inspiratory esophageal pressure; $Ppeak_{cw}$: peak inspiratory pressure of the chest wall; $Ppeak_{l}$: lung peak pressure; $Ppeak_{rs}$: peak inspiratory pressure of the respiratory system; RR: respiratory rate; SVV: stroke volume variation; TPP_{ei}: end-inspiratory transpulmonary pressure; TPP_{ee}: end-expiratory transpulmonary pressure; TPP_{elast}: elastance-derived transpulmonary pressure; VT_{insp}: inspiratory tidal volume.

Supplementary Fig. A1 and A2

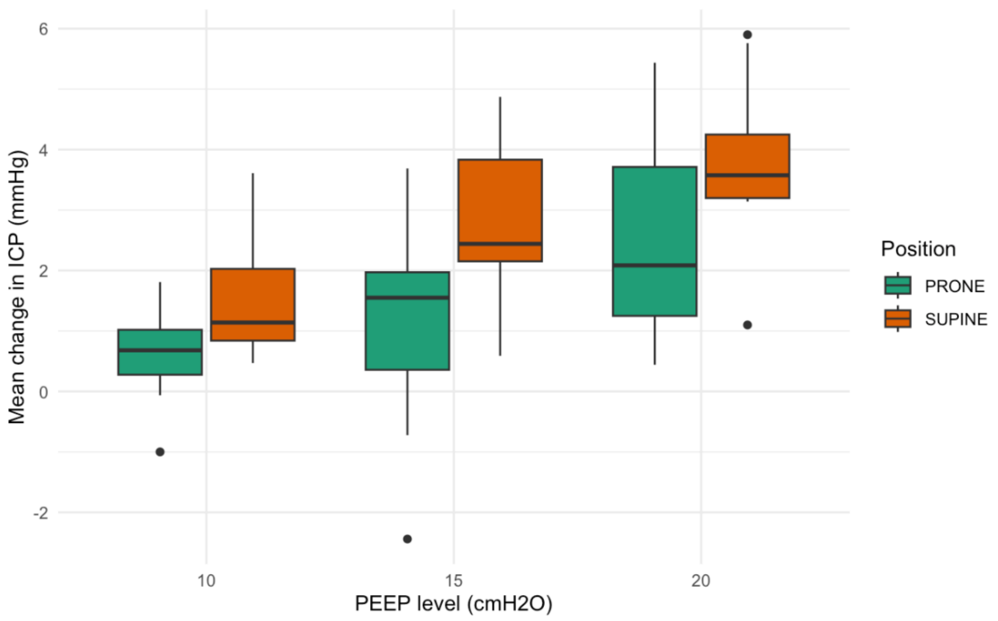

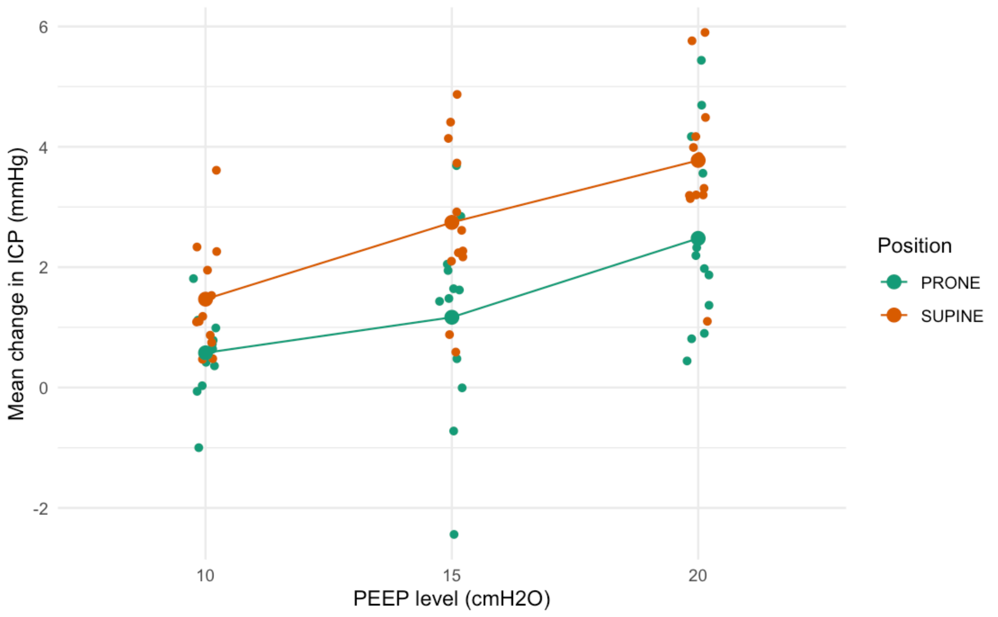

***Figure A1 and A2** Mean change in intracranial pressure (ICP) at different positive end-expiratory pressure (PEEP) levels in prone and supine positions visualized with boxplot (B1) and interaction plot (B2). Two-way ANOVA showed no significant interaction between PEEP*

levels and position (p=0.66). PEEP and position each significantly affected the change in ICP, regardless of PEEP level (p<0.001).

Supplementary Fig. A3

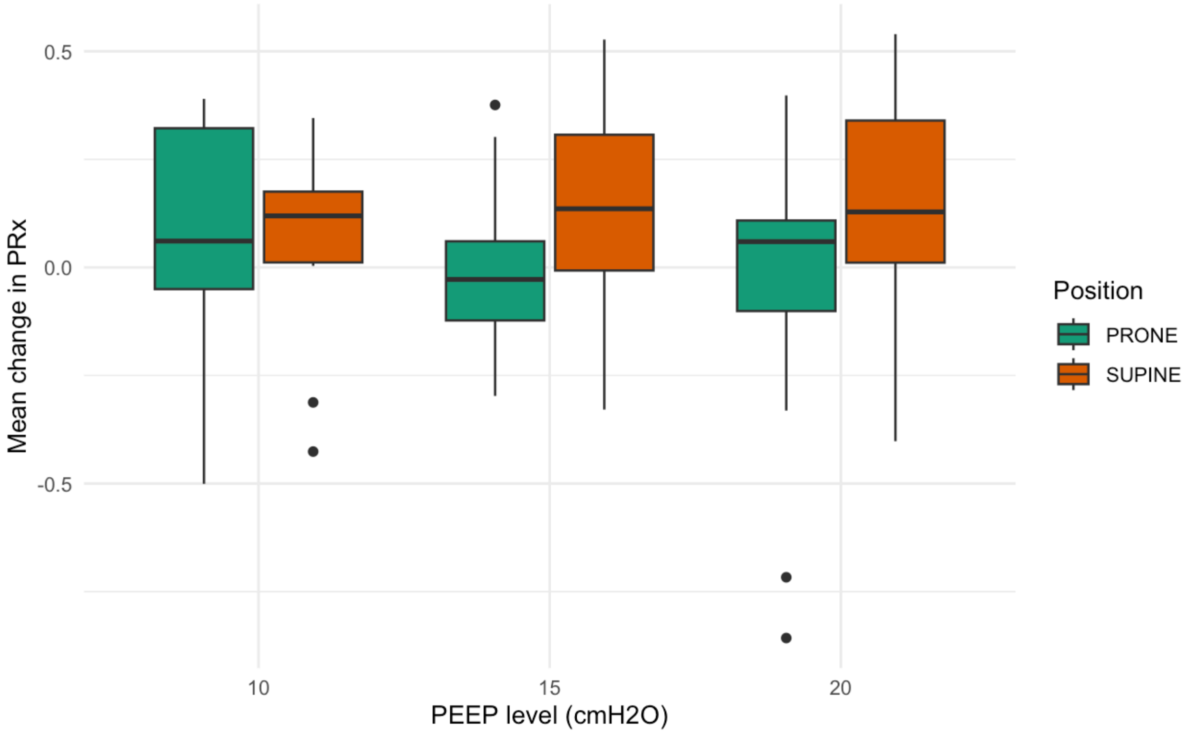

Figure A3 *Mean change in Pressure Reactivity Index (PRx) at different positive end-expiratory pressure (PEEP) levels in prone and supine positions. The PEEP increase did not significantly change mean PRx compared to the baseline value (5 cmH$_2$O).*

Supplementary Fig. B.

Correlation plots for baseline variables against mean change in intracranial pressure (ICP)

Supplementary Fig. B1 Ers

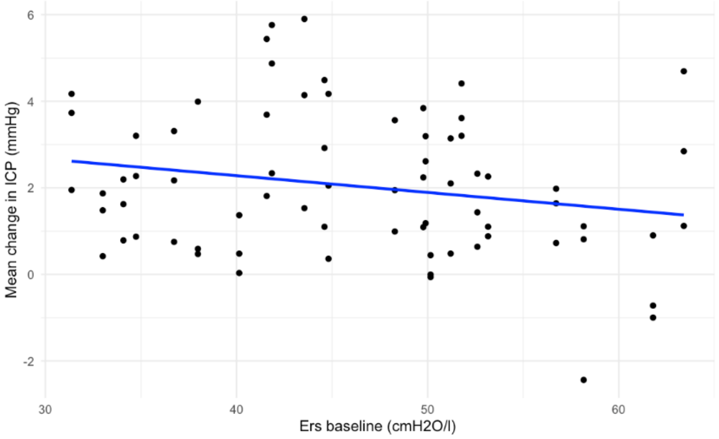

Figure B1 *Mean change in intracranial pressure (ICP) versus baseline elastic resistance (Ers).*

Significant negative correlation (p=0.024, r=-0.21).

Supplementary Fig. B2 TPP_{ee}

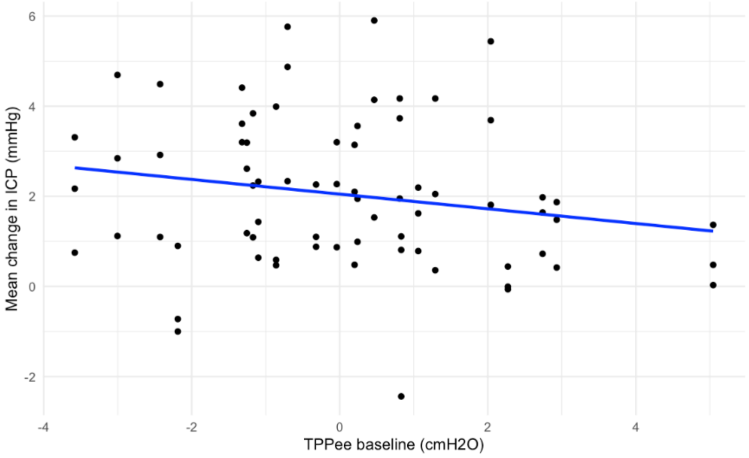

Figure B2 *Mean change in intracranial pressure (ICP) versus baseline transpulmonary pressure at end-expiration (TPPee).*

Significant negative correlation (p=0.009, r=-0.20)

Supplementary Fig. B3 TPP_{ei}

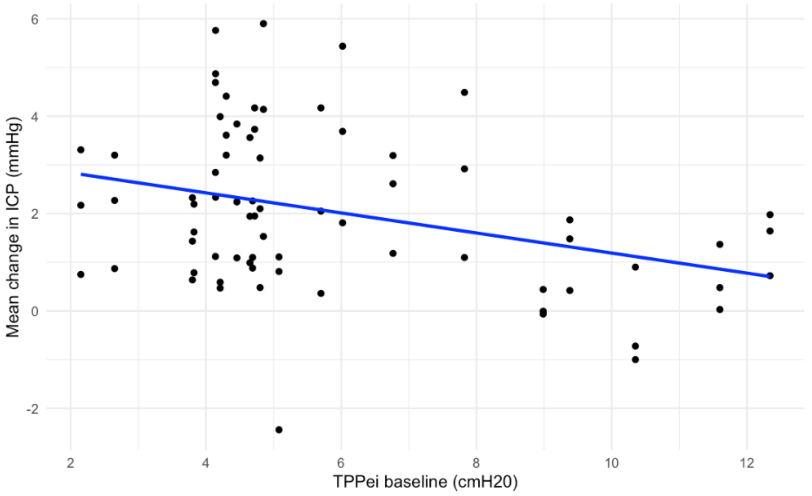

Figure B3 *Mean change in intracranial pressure (ICP) versus baseline transpulmonary pressure at end-inspiration (TPPei).*

Significant negative correlation (p=0.006, r=-0.34)

Supplementary Fig. B4 CVP

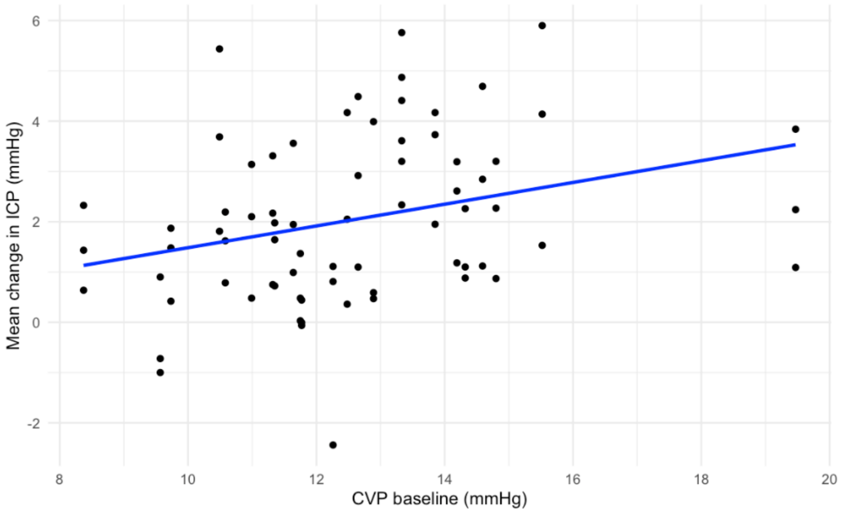

Figure B4 *Mean change in intracranial pressure (ICP) versus baseline central venous pressure (CVP).*

Significant positive correlation (p=0.0032, r=0.30).

Supplementary Fig. B5 ICPmean

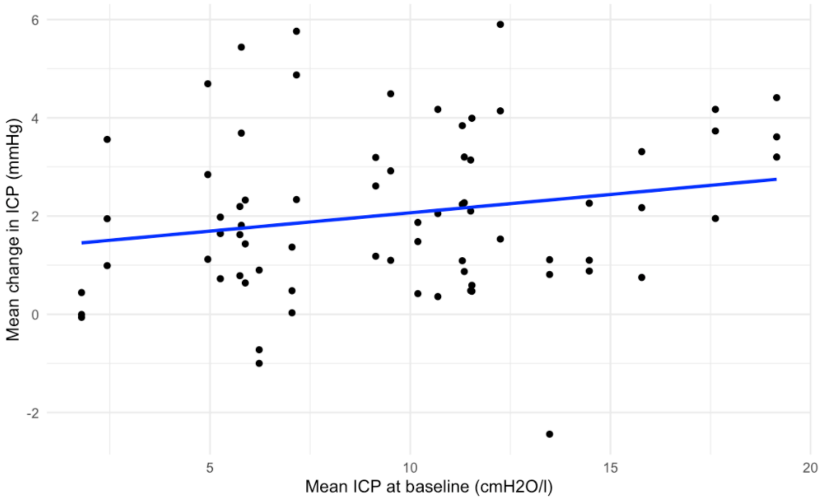

Figure B5 *Mean change in intracranial pressure (ICP) versus mean ICP at baseline.*

Significant positive correlation (p=0.012, r=0.20).
